# Simplification Is Not Dominant in the Evolution of Chinese Characters

**DOI:** 10.1162/opmi_a_00064

**Published:** 2022-12-02

**Authors:** Simon J. Han, Piers Kelly, James Winters, Charles Kemp

**Affiliations:** Melbourne School of Psychological Sciences, University of Melbourne, Parkville, Australia; Department of Archaeology, Classics and History, University of New England, Armidale, Australia; School of Collective Intelligence, Mohammed VI Polytechnic University, Rabat, Morocco

**Keywords:** Chinese characters, cultural evolution, communicative efficiency, complexity, distinctiveness

## Abstract

Linguistic systems are hypothesised to be shaped by pressures towards communicative efficiency that drive processes of simplification. A longstanding illustration of this idea is the claim that Chinese characters have progressively simplified over time. Here we test this claim by analyzing a dataset with more than half a million images of Chinese characters spanning more than 3,000 years of recorded history. We find no consistent evidence of simplification through time, and contrary to popular belief we find that modern Chinese characters are higher in visual complexity than their earliest known counterparts. One plausible explanation for our findings is that simplicity trades off with distinctiveness, and that characters have become less simple because of pressures towards distinctiveness. Our findings are therefore compatible with functional accounts of language but highlight the diverse and sometimes counterintuitive ways in which linguistic systems are shaped by pressures for communicative efficiency.

## INTRODUCTION

A common expectation about the world’s writing systems is that their symbols evolve to become simpler over time. This idea is compatible with a broader literature on signed, spoken and written language that emphasizes ways in which linguistic systems are shaped by the need to support efficient communication (Gibson et al., 2019; Keller, 2005; Kirby et al., 2015; Tamariz & Kirby, 2015; Zipf, 1949). Just as speakers simplify and shorten words in order to communicate with greater efficiency (Kanwal et al., 2017), written symbols undergo comparable transformations that remove superfluous graphical details and reduce visual complexity (Changizi & Shimojo, 2005; Dehaene, 2009; Garrod et al., 2007; Kelly et al., 2021; Pauthier, 1838; Trigger, 2003).

**Figure 1.**
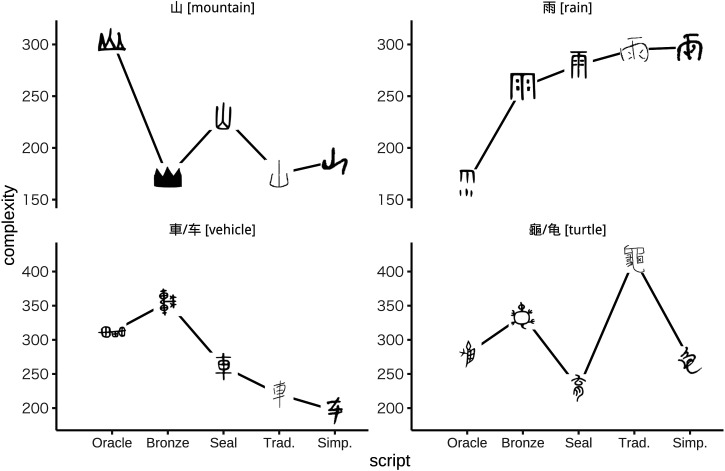
**Changes in four pictographic Chinese characters over time.** Relative to oracle bone forms, traditional forms for 山 [mountain] and 車 [vehicle] are simpler but traditional forms for 雨 [rain] and 龜 [turtle] are more complex. The y-axis of each panel shows perimetric complexity. Our dataset includes an average of around 50 variants of each character for each script but only the median complexity variant is plotted here.

As the world’s only primary script still in continuous use, Chinese writing is regularly invoked as a compelling illustration of graphic simplification over historical time. Classical and modern Chinese philologists have long commented on processes of change and simplification in the Chinese script (for historical overviews see Behr (2005), Bottéro (1998), and Erlman (1990)), and European scholars continued this intellectual trend (Pauthier, 1838; Warburton, 1741). For example, H. J. Klaproth suggested that through regular tracing the once-iconic Chinese characters became more “abbreviated and cursive” as the features of their images began to “blur and disappear” resulting in a kind of shorthand (Klaproth, 1832). Consistent with this view, for the past 350 years scholars have produced diagrams of Chinese characters that depict a straightforward linear sequence from iconic pictures towards abstract signs (Garrod et al., 2007; Kircher, 1654; Klaproth, 1832; Martini, 1658; Pauthier, 1842), and two similar sequences are shown on the left side of Figure 1. The idea of simplification remains prominent in modern literature on the Chinese script, and in the literature on cultural evolution (Fay et al., 2013; Garrod et al., 2007). For example, Woon (1987, p. 1) writes that “in the past 4000 years, Chinese characters have always been in a process of simplification,” and Tsien (1962, p. 183) states that the Chinese script “is evolutional from complex to simple construction.” Qiu (2000, p. 48) acknowledges exceptions to this general trend, but writes that “although there are cases of certain forms becoming more complex, they pale in significance when compared with the importance of simplification.”

Although the idea that characters typically simplify is intuitive, it should not be taken for granted. Linguistic systems are shaped by multiple pressures—some of these forces reinforce each other but others act in opposite directions (Haiman, 2010). If we consider a single character in isolation, reducing the complexity of the character may make it easier to read and write. Yet if we consider the entire inventory of characters, reducing visual complexity may make the characters harder to distinguish from each other (Pelli et al., 2006; Wiley & Rapp, 2019). Even a randomly-generated inventory of symbols may be distinctive enough if the inventory is small, but distinctiveness is harder for large symbol inventories to achieve, and may have become especially relevant to written Chinese as the size of the character inventory has grown over time (Chang et al., 2016; Miton & Morin, 2021). If simplicity and distinctiveness trade off against each other, then simplification over time no longer appears to be inevitable, and two additional hypotheses must be considered. If the relative weights of these factors shift in favour of distinctiveness over time, then it is possible that character complexity will increase, as has occurred for the examples on the right of Figure 1. Alternatively, if simplicity and distinctiveness remain in equilibrium, it is possible that character complexity will remain steady over time. Some support for this final hypothesis is provided by the recent work of Miton and Morin (2021) who analyzed a phylogeny including more than a hundred scripts and report that descendant scripts show no general tendency to either increase or decrease in complexity relative to ancestor scripts.

To adjudicate between these hypotheses, we examine the evolutionary trends of the Chinese script over the course of its recorded history. We view Chinese writing as a large natural experiment in which countless readers and writers over thousands of years have shaped its graphical landscape in ways that reflect the fundamental pressures acting upon the evolution of writing systems more broadly. By leveraging computational methods at scale, we attempt to clarify how and why the Chinese writing system has changed in visual complexity over time.

## METHOD & RESULTS

We began by collecting 38,066 images of historical Chinese characters from a popular Chinese etymology website called hanziyuan.net. Hanziyuan includes forms from three key historical scripts: oracle bone script (甲骨文), bronze script (金文), and small seal script (小篆書). The oldest surviving examples of the Chinese script are oracle bone inscriptions from the Shang dynasty (ca. 1600–1046 bce). These texts were incised on ox scapulae and turtle plastrons and used in divination ceremonies. Bronze script appears on objects cast in bronze including vessels, bells and tripods, and was often produced by writing on the soft clay moulds used to cast these objects. Early bronze inscriptions date from the Shang dynasty and are coeval with oracle bone inscriptions, but bronze script is most characteristic of the Western Zhou (1046–771 bce) and Spring and Autumn (660–476 bce) periods. After these periods a variety of scripts were used by the independent states of the Warring States period (476–221 bce). The country was subsequently unified under the Qin dynasty (221–206 bce), and small seal script was the official standard script during this dynasty. To complete our dataset we added handwritten modern characters from two scripts: traditional script (正體字) (Chen, 2020), which is used today in Taiwan, Hong Kong, and Macau and by parts of the Chinese diaspora, and simplified script (简化字) (Liu et al., 2011), which replaced the traditional script in mainland China. As described later, we also analyzed printed modern characters, but chose to focus on handwritten modern forms for maximum comparability with oracle bone forms.

Although our dataset includes more than half a million images of Chinese characters it provides an incomplete picture of the great diversity of historical Chinese scripts. By necessity we are constrained to work with sign forms that have survived in the historical record and can be dated to a period; it is not possible to probe the scope of written traditions that left no trace or are yet to be uncovered. Even among surviving materials, entire scripts are missing from our data, including scripts used during the Warring States period (Park, 2016) and the clerical script widely used during the Han dynasty (206 bce–220 ce). Further, within any period there may be substantial differences between the standard form of a character and a range of informal variants (including cursive forms), and our dataset focuses on standard forms. Despite these limitations, our data seem sufficiently rich to determine whether or not the evolution of Chinese characters shows a general tendency towards simplification, as we explain in more detail below.

The full set of images includes representatives of 3,889 distinct characters. This set includes all characters that appear either on hanziyuan.net or in one of our modern handwritten data sets, and also in the Chinese Lexical Database (CLD) (Sun et al., 2018). We focus on characters from the CLD because our analyses draw on information including character frequency that is included in this database. For each of the 3,889 characters in our dataset, we have up to 291 images of its variants from each script. When a character has multiple variants within a single script, the complexity of a character is defined as the median complexity across all of these variants. Images from all sources underwent the same preprocessing steps to control for size and stroke thickness, and full details can be found in the supplementary material.^1^

Following previous studies (Garrod et al., 2007; Kelly et al., 2021), we define the visual complexity *C* of an image as its perimetric complexity (Arnoult & Attneave, 1956; Pelli et al., 2006):

$$C=\frac{P^{2}}{4\pi A},$$ (1)

where *P* is the sum of the interior and exterior perimeters of the image, and *A* is its area. Perimetric complexity has been shown to predict several aspects of human perception including the efficiency, accuracy and speed of recognizing letters and characters from multiple scripts including modern Chinese (Chang et al., 2016; Pelli et al., 2006; Wang et al., 2014; Wiley et al., 2016; Zhang et al., 2007). Other complexity measures are possible, including the number of black pixels in an image, the length of an image’s description in a standardized representation language, and measures related to writing such as the number of strokes in a character and the approximate time taken to write a character. Previous work suggests that alternative measures like these are highly correlated both with perimetric complexity and with each other (Wang et al., 2014; Zhang et al., 2007), and we report similar results in the supplementary material. The substantial correlations between all of these measures suggest that our conclusions are probably robust to the choice of complexity measure.

### Changes in Complexity Over Time

**Figure 2.**
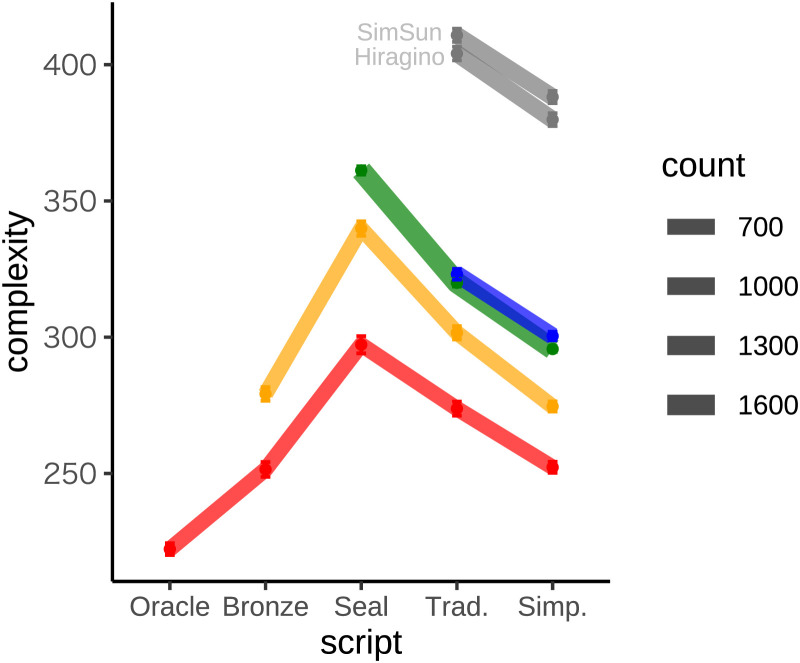
**Complexity over time of characters grouped according to their first appearance in our dataset.** The first stream (red) includes characters for which we have at least one oracle bone form, and the bronze, seal and traditional streams are shown in yellow, green and blue respectively. Grey lines show results for the traditional stream based on characters printed in two fonts. Line thickness is proportional to the number of characters included in each stream, and error bars (which are small and therefore difficult to see) show the standard error of the mean.

Figure 2 shows how character complexity has changed across the five scripts in our analysis. Each character has been assigned to one of four streams depending on the script in which it first appears in our dataset: for example, the oracle stream includes all characters for which we have an oracle bone form. Figure 2 suggests that characters tend to increase in complexity up to seal script and subsequently become less complex. To confirm the changes in complexity suggested by Figure 2, we used the brms package (Bürkner, 2017) to run a Bayesian mixed effects regression with script as a predictor of complexity, and included character as a random intercept and a random slope for script. The 95% credibility intervals for the coefficients that capture differences between successive scripts all exclude zero, suggesting that the two increases in complexity up to seal script and the two subsequent decreases are all statistically reliable. Figure 2 also includes results for modern characters printed in two fonts. Complexity scores are substantially higher for printed than for handwritten forms, but regardless of whether we consider printed or handwritten versions of modern characters, we find that traditional and simplified forms are both more complex than their oracle counterparts.

Figure 2 reveals two distinct ways in which character complexity has increased through time. First, the oracle and bronze streams both increase in complexity up through seal script, suggesting that individual characters often increase in complexity. Second, the characters in each successive stream tend to be more complex than characters in previous streams, suggesting that there is a tendency for new characters added to the inventory to be more complex than existing members. Because our dataset is missing many forms, and because forms from earlier scripts are more likely to be missing, this finding must be interpreted with caution. For example, thousands of known oracle bone forms are missing from our dataset because they have never been deciphered.

Although our results reveal a net increase in complexity over 3000 years, we do find evidence of simplification from the seal script on. Scholars often suggest that the transition between seal script and modern characters involved a process of simplification (Schindelin, 2019), and our results for handwritten (but not printed) traditional characters support this view. The simplified script was specifically designed to reduce the visual complexity of written Chinese (Pan et al., 2015), and as expected our results for both handwritten and printed characters confirm that simplified forms are less complex than traditional forms. Our results therefore provide partial support for the standard view that writing systems are shaped by forces that tend towards simplification, but challenge the idea that these forces have been dominant over the history of the Chinese script.

Although Figure 2 suggests that modern forms tend to be more complex than oracle forms, it is possible that some kinds of characters defy this overall trend. Characters with iconic origins, characters with small numbers of components, and high frequency characters all seem like especially good candidates for simplification. We now consider each of these subclasses in turn, and in all three cases we report consistent evidence for increases in complexity over time.

**Figure 3.**
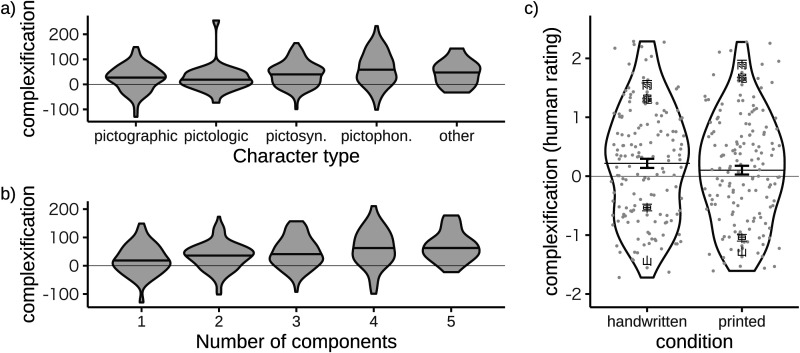
**Changes in complexity between oracle and traditional forms.** (a)–(b) Complexity changes for characters of different types and with different numbers of components. The thin line at zero represents no change in complexity, and the interior of each violin plot shows the median. (c) Human ratings of the relative complexity of oracle bone and traditional forms (handwritten or printed). Each of the jittered grey points shows ratings for one of 155 pictographic characters: of the characters shown in Figure 1, 山 and 車 are judged to simplify and 雨 and 龜 are judged to become more complex. Characters on the zero line have oracle and traditional forms that are rated as equally complex, and the error bars show the standard error of the mean.

Informal discussions of the simplification of Chinese often refer to examples involving characters like 車 [vehicle] (see Figure 1) and 馬 [horse] that originated from detailed illustrations of animals and other concrete natural elements (Norman, 1988; Qiu, 2000). Because iconic images tend to be complex, it is natural to think that unnecessary detail should be shed over time (Norman (1988), although see Miton and Morin (2019)), and this intuition probably accounts for the widespread assumption that Chinese characters typically simplify. To test this intuition we drew on the character classifications available in the CLD. Characters classified as pictographic originate from iconic forms, and pictologic characters are similar but more symbolic in nature. Pictosynthetic characters are combinations of multiple pictographic characters, and pictophonetic characters are combinations of phonetic and semantic components. The fifth class (other) is a catch-all, and each character is assigned to exactly one class. To see the strongest possible differences between oracle bone and modern forms we treat traditional characters as representatives of the modern era, and Figure 3a suggests that characters from all five classes increased in complexity between the oracle bone and traditional scripts. The analysis includes only characters that are present in our dataset for both scripts, and the y-axis (complexification) shows the difference in perimetric complexity between the two scripts. The supplementary material includes analyses which suggest that the increase in complexity for each class is statistically reliable. We therefore conclude that the net increase in complexity between oracle bone and traditional forms summarized by Figure 2 applies to many kinds of characters, including those with iconic origins.

Because our finding that pictographic characters have increased in complexity challenges a common view about the evolution of writing systems, we developed a preregistered behavioral experiment to address the concern that this finding may be an artifact of perimetric complexity.^2^ The experiment asked 400 participants who were not fluent in Mandarin, Cantonese or Japanese to rate the relative complexity of 155 pairs of forms. The characters used were identical to the 155 pictographic characters assigned to the pictographic group in Figure 3a. In the handwritten condition, the traditional forms were drawn from the same set of handwritten characters analyzed in Figure 3a, and in the printed condition the traditional forms were shown in Hiragino Sans GB. Figure 3c shows that on average traditional forms were rated as more complex than oracle bone forms in both the handwritten and printed conditions. A set of preregistered statistical tests supported this conclusion for handwritten but not printed characters. Full details are available in the supplementary material, and taken overall the results support the conclusion that pictographic characters have traditional forms that are more complex than their oracle bone forms. In addition, the experiment provides some evidence that perimetric complexity is an adequate complexity measure for our purposes.

One way for a character to increase in complexity is to acquire new components. Characters with modern forms consisting of a single component only (e.g., 車 [vehicle]) may therefore be especially likely to show evidence of simplification. We used data from the Chinese Characters Decomposition (CCD) project to sort our dataset into characters with different numbers of components (Wikimedia Commons, 2021). The CCD project is based on simplified characters and provides decompositions that are purely graphical rather than etymological. Even so, these data provide a useful way to distinguish characters with different numbers of components. Figure 3b shows that even characters with a single component have become more complex over time. Increases in complexity, however, tend to be greater for characters with multiple components than for single component characters.

One possible reason for simplification is that writers sometimes cut corners and simplify when reproducing a character. On this account, the characters written most frequently should be most likely to simplify. This hypothesis is consistent with Zipf’s law of brevity, which states that frequently used linguistic units tend to be especially simple, and with a body of related work that has explored how language is shaped by efficiency considerations (Bentz & Ferrer-i Cancho, 2016; Zipf, 1949). We tested this hypothesis by using character frequencies from the CLD and assuming for simplicity that CLD frequencies (which are based on modern data) are also representative of frequencies for earlier scripts. Some characters are components of other characters, and we define the adjusted frequency of a character as the number of times it is written per million characters, either in isolation or as part of another character. We sorted our characters into six frequency bins using a logarithmic scale of base ten, and compared average character complexity in each bin both within and across scripts.

**Figure 4.**
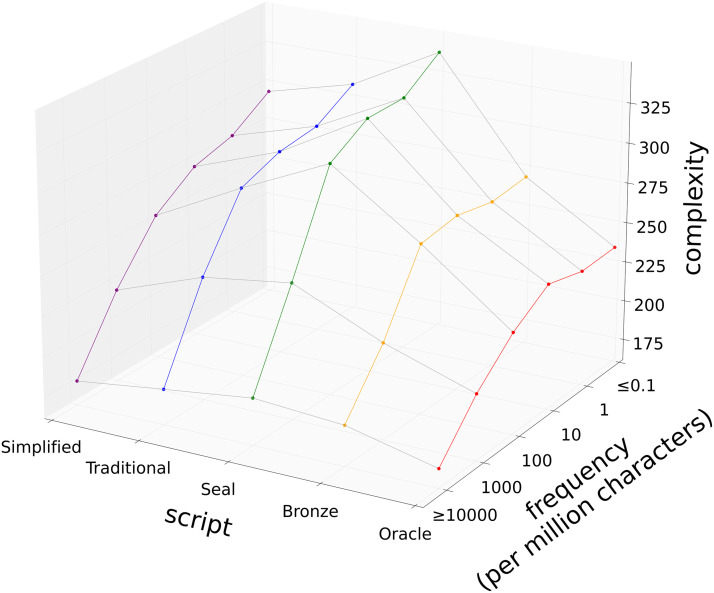
**Complexity over time for characters in six frequency bins.** The labels of the frequency bins represent counts per million characters. Only characters for which we have an oracle bone form have been included.

Figure 4 shows that characters within each frequency bin show parallel changes in complexity over time. This result indicates that even the most frequently used characters do not simplify over time. Although characters in all frequency bins have higher traditional complexities than oracle bone complexities, within each script frequently used characters tend to be simpler. This result is consistent with Zipf’s law of brevity, and suggests that Chinese characters are indeed shaped by efficiency considerations. Figure 4 also reveals that changes in complexity over time are modulated by frequency, and that frequently used characters tend to show smaller increases in complexity up to seal script and smaller decreases in complexity thereafter. High frequency, however, is evidently not sufficient to produce simplification overall. A statistical analysis supporting all of these conclusions is presented in the supplementary material.

Our analyses so far provide consistent evidence that modern characters are more complex than oracle forms, and suggest that pictographic characters, single-component characters and high frequency characters are not exceptions to this general trend. These results came as a surprise to us, and led us to consider possible reasons why complexity may have increased over time. The next section introduces two potential explanations, both of which invoke evolutionary pressures in favor of distinctiveness. Both explanations seem plausible to us, but we acknowledge that we do not have strong evidence for either one.

### Complexity and Distinctiveness

The expectation that characters tend to simplify can be informally motivated by the idea that writing systems increase in communicative efficiency over time. Simplicity, however, is just one relevant dimension, and communicative efficiency is best conceptualized as a near-optimal trade-off between several competing dimensions (Kemp et al., 2018). We focus here on the trade-off between simplicity and distinctiveness, or the ease with which characters can be distinguished from each other (Wiley & Rapp, 2019). If simplicity and distinctiveness are inversely related—that is, if more complex characters are also more distinctive—then pressures toward distinctiveness could help to explain why complexity has increased over time. The character inventory could remain communicatively efficient at all stages of this process as long as simplicity is always maximized for the current level of distinctiveness.

Measuring distinctiveness is challenging, and to our knowledge there is no standard approach in the literature. We therefore developed our own distinctiveness measure using a convolutional neural network (CNN) trained to classify handwritten Chinese characters. The results emerging from this measure are suggestive, but as we discuss later the measure is subject to some important limitations. We therefore view our distinctiveness analyses as a tentative initial exploration that should be revisited and extended in future as improved distinctiveness measures become available.

Our measure is motivated in part by previous work suggesting that the internal representations generated by CNN classifiers provide a good account of human similarity judgments (Peterson et al., 2018). In our case, the CNN is a GoogLeNet architecture trained on a large database of simplified characters (Zhong et al., 2015). To make our character images maximally comparable to the images on which the CNN was trained, we included an extra image processing step that increased the stroke width of each character. Passing an image through the network generates an activation vector over each layer, and we took the activation over the final fully connected layer as the representation for each character. Distinctiveness can then be defined as the average Euclidean distance between a character and its closest 20 contemporary neighbours. The neighborhood size of 20 is based on previous work on orthographic similarity that uses the same definition of distinctiveness but different underlying representational spaces (Sun et al., 2018; Yarkoni et al., 2008). In cases where our data include multiple images for a specific character in a specific script, we treat the median complexity image as the definitive variant of the character.

**Figure 5.**
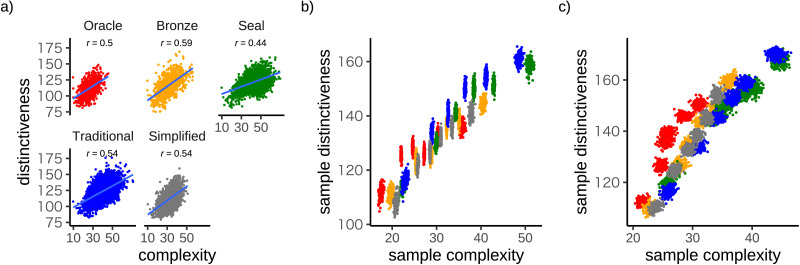
**Visual complexity trades off against distinctiveness.** (a) Relationship between distinctiveness and perimetric complexity for each script in our dataset. Complexity values are lower here than in Figure 2 because the character images in this analysis have thicker strokes. (b)–(c) Relationship between average distinctiveness and average complexity for miniature 50-character inventories. For each script, samples were drawn from 6 complexity bins (panel b) or 6 distinctiveness bins (panel c).

We used the distinctiveness measure just introduced to explore whether complexity and distinctiveness trade off against each other. Figure 5a shows that character complexity and distinctiveness are positively correlated within each script. This result suggests that complexity and distinctiveness trade off at the level of individual characters, and that individual characters may need to become more complex in order to become more distinctive. To explore whether a similar trade-off applies at the level of entire systems of characters, we repeatedly sampled miniature systems of 50 characters and asked whether systems with higher average distinctiveness also tend to be higher in average complexity. We generated samples separately for each script using two distinct sampling strategies. Figure 5b is based on sorting the characters in each script into 6 complexity bins (low complexity to high complexity), and then generating 200 random samples within each bin. Figure 5c used a similar approach except that the bins were based on distinctiveness rather than complexity. In both cases, average complexity and average distinctiveness were correlated, suggesting that complexity and distinctiveness trade off at the system level.

**Figure 6.**
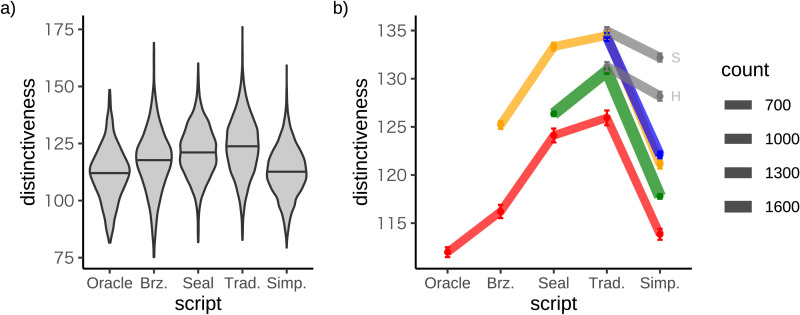
**Changes in distinctiveness over time.** (a) Distinctiveness distributions for each script. The horizontal black lines show medians. (b) Change in distinctiveness for characters grouped according to their first appearance in our dataset. Within each stream distinctiveness is always computed with respect to the same set of characters. Error bars show the standard error of the mean. Grey lines show traditional streams that include characters printed in SimSun (S) or Hiragino Sans GB (H).

Next we considered how distinctiveness has changed over time, and Figure 6a shows a steady increase in distinctiveness up to the traditional script. To control for inventory size, Figure 6b shows distinctiveness computed with respect to the same set of characters over time. The oracle stream includes all characters that first appear in the oracle bone script and that are attested in all subsequent scripts, and the bronze, seal and traditional streams are defined analogously. Direct comparisons of distinctiveness between streams (e.g. bronze vs seal) are not possible because the streams have different numbers of characters, and the key question is how distinctiveness changes over time within each stream. For all streams, Figure 6b reveals that distinctiveness increases up to the traditional script and then falls.

For comparison with the results for handwritten characters, Figure 6b includes versions of the traditional stream for characters printed in two fonts. Because handwritten characters are produced by writers who desire to minimize writing time, we expected distinctiveness scores to be lower for handwritten than for printed characters. This finding emerges for simplified characters, but for traditional characters distinctiveness is lower for characters printed in Hiragino Sans GB than for handwritten characters. A second unexpected result is that across both traditional and simplified scripts, distinctiveness is substantially lower for Hiragino than for SimSun. A possible explanation for both results is that our distinctiveness measure is overly sensitive to stylistic differences (e.g. whether or not a font includes serifs) that are of limited interest for our purposes.

Our distinctiveness measure is subject to another important limitation which means that the results in Figure 6 should be taken as suggestive but not conclusive. The neural network that we used was trained on simplified characters, and may be relatively poor at distinguishing between oracle bone forms largely because they are qualitatively different from the simplified forms in the training set. This concern does not affect the finding that distinctiveness and visual complexity appear to trade off within each script (Figure 5), but does affect our comparisons across scripts (Figure 6). Future research can potentially address this concern by supplementing our distinctiveness results with similar analyses based on a network trained on oracle bone forms.

Establishing a causal account of historical change does not seem possible given the data available to us, but we offer two plausible explanations of the finding that complexity and distinctiveness have both increased through time. The first explanation holds that distinctiveness is the driving factor, and that an increase in distinctiveness has caused complexity to increase. Distinctiveness is especially relevant to readers, who must distinguish each character viewed from possible alternatives, and may have become increasingly important as the relative balance between readers and writers has shifted over time. In the modern era, a character that is written, carved or inscribed once can be read by an audience of millions, and it seems plausible that the average audience size for each act of writing has steadily increased over time.

The second possible explanation holds that neither complexity nor distinctiveness is the driving factor, but that both have been influenced by a third factor—the dramatic expansion of the Chinese character inventory over time. When new characters are added, distinctiveness must remain above some threshold in order for the script to remain usable. If most of the simple forms are already taken, new characters will have to be relatively complex in order to maintain distinctiveness above this threshold, which means that average complexity will increase over time. In principle, it may be possible to add new characters while holding average distinctiveness constant, but this possibility may be unachievable if new characters must created by reusing components of existing characters. As a result, it is possible that increasing inventory size inevitably requires increases in both complexity and distinctiveness.

Our two possible explanations are not mutually exclusive, and it is possible that the balance between complexity and distinctiveness has shifted over time and that the expansion of the character inventory has driven increases in both complexity and distinctiveness. These two possibilities, however, are conceptually distinct, and the first could apply even if the size of the character inventory were held constant. Although both explanations seem plausible to us, the second seems likely to carry more weight because the expansion of the character inventory is such a striking development in the history of Chinese characters. To understand this development in more detail, future studies could simulate different hypothetical strategies for generating novel characters over time, and could directly test the idea that the only feasible strategies lead to increases in average complexity and average distinctiveness.

## DISCUSSION

Writing systems are often thought to simplify over time, but we found that the visual complexity of modern Chinese characters has increased relative to oracle bone forms. This increase in complexity has occurred at the level of individual characters and at the level of the entire inventory, whose average complexity has been increased by the addition of relatively complex characters. The iconicity of early Chinese characters has not stood in the way of this process, with early iconic forms complexifying over time even as they become more abstract. High frequency, likewise, is not enough to protect against increases in complexity. When we look beyond the popular examples brought forward by proponents of simplification, we see that for every intuitive example of simplification (e.g. left side of Figure 1), there are many other examples of complexification occurring instead (right side of Figure 1).

A plausible explanation for our results is that writing systems, just like languages, are subject to multiple competing pressures, including a pressure for distinctiveness that trades off against a pressure for visual simplicity. Future work can aim to measure and evaluate additional factors that influence the ease of reading, writing and learning characters. For example, the compositionality of the system (Myers, 2019), or the extent to which characters are composed out of standardized recurring elements will affect the ease with which characters can be learned. Ease of learning probably trades off against visual simplicity: for example, Hannas (1988, p. 210) points out that 鑫 is high in visual complexity but relatively easy to learn because it repeats a single element three times.

The compositionality of a system can potentially be formulated using a setwise complexity measure that assesses the complexity of entire systems of characters. One such measure, for example, defines the complexity of a set as the length of the minimal description of all characters in the set. If the characters in the set are all built from a small library of components, then the minimal description would involve describing each component then specifying how the components are combined to form characters. Although our results suggest that the average visual complexity of individual characters in the Oracle stream has increased over time, the setwise complexity of these characters may well have decreased as the writing system has become more compositional. Testing this idea would probably require a sophisticated computational approach that draws on techniques from the literature on computer vision in order to capture elements that recur across sets of handwritten characters.

### Reconciliation With Prior Work

At first sight, our finding that Traditional forms are more complex than Oracle forms seems directly incompatible with earlier claims about the evolution of writing and of the Chinese script in particular. Our disagreement with prior research, however, is perhaps less fundamental than it seems. To our knowledge, previous studies have not directly measured changes in the visual complexity of Chinese characters over time, which means that our findings do not conflict with any specific empirical results from the literature. The conflict is rather with general claims about how the Chinese script has developed over time.

In the literature on written Chinese, “simplification” has been used in a range of different ways. In discussions of the shapes of individual characters, simplification is broadly used to refer to a bundle of changes that includes a progression away from pictorial forms and towards more abstract symbols in addition to changes in visual complexity. Simplification has also been used to refer to increases in consistency across tokens of a single character, and to increases in stylistic consistency across an entire script, including the development of a repertoire of standard strokes (Qiu, 2000). Because simplification has been used to label so many different kinds of changes, many previous ideas about simplification remain intact despite our findings about changes in visual complexity over time.

If we focus on visual complexity in particular, experimental work (Garrod et al., 2007) and a prior analysis of the Vai script (Kelly et al., 2021) both suggest that written symbols tend to be relatively complex when first created but become simpler as they are repeatedly used. These results led us to anticipate similar changes in written Chinese, but in retrospect we see two important differences between our work and the studies of both Garrod et al. (2007) and Kelly et al. (2021). First, both previous studies trace the evolution of symbols from their moment of birth onwards, but the earliest forms in our analysis are drawn from a time at which Chinese characters had already been in use for hundreds of years. The historical record does not reveal what the very first Chinese characters looked like, and it is possible that the earliest stages in the development of the script were characterized by decreases in visual complexity. Second, both previous studies considered symbol inventories that were relatively stable in size over time, but we considered a system that has significantly increased in size. It is possible that simplification is typical when the size of an inventory remains constant, but that as an inventory increases in size, complexification becomes necessary in order to hold distinctiveness at an acceptable level.

### Limitations and Caveats

Although our work highlights the idea that the graphic dimension of writing is shaped by general functional principles, our results are coloured by the historical and material context in which written Chinese developed. Our dataset covers a period of approximately 3,000 years; in this time, the characters that we study have transitioned from brushed and etched signs to digital fonts typed onto computer screens. There is no doubt that the epigraphic technology available in a given period has conditioned the degree of complexity that the script could tolerate. Just as the change from a reed stylus to a wedge-tipped stylus in mid-third millennium Mesopotamia introduced a more compact and consistent style of cuneiform, in China a transition from bone carving to the use of soft-clay impressions, for example, would have altered the parameters of graphic possibility (Demattè, 2010; Škrabal, 2019).

The social functions of different scripts are also likely to influence their relative complexities. For example, scripts used informally may tend to be simpler than scripts used for official documents, and ornamental scripts used for display purposes may be especially complex. Historical precedent and contact with other graphic traditions are also factors that bear consideration in any examination of script change. However, few palaeographers subscribe to a strictly deterministic view of script evolution whether in terms of scribal media, social function or contact. For example, technological shifts in the production of the Vai script, from reed pens to modern pencils and digital fonts, do not account for any substantial changes in visual complexity, nor indeed did standardisation campaigns or shifts in genre (Kelly et al., 2021). The reality that the Egyptian hieroglyphic script was transformed into the much simpler hieratic script is in no way negated by the continued and concurrent use of the hieroglyphic script for monumental display. In short, we maintain that the material and ideological circumstances of a writing system are informative but do not overwhelm the dynamics of change brought about by actual use, including reading, writing and inter-generational transmission.

The most recent phase in the history of written Chinese concerns the simplified script reform of 1956, when China’s Ministry of Education replaced a core set of characters with simplified versions. This politically-motivated reform could hardly be characterised as a subtle invisible-hand process, yet it is still part of the bigger story of script change and demands its own explanation. After all, deliberate acts of simplification have taken place several times in the history of the script (for an early example see Semedo (1655, p. 43). Two nation-wide campaigns, in 1935 and 1977, failed abysmally and even the 1956 reform had its limitations. Despite affecting only about half of the inventory, the over-simplification of certain characters nonetheless introduced unintended reading difficulties (Pan et al., 2015) suggesting that the pull towards distinctiveness is formidable even in the face of heavy reform.

### New Directions

Our results suggest that simplification is not the dominant trend in the evolution of Chinese characters, but additional work is needed to determine the extent to which complexification has occurred. One pressing need is for a dataset that includes more scripts than the five analyzed here. Some of these scripts will correspond to subdivisions of the oracle-bone and bronze scripts considered here. For example, oracle-bone sources have been organized into five periods (Dong, 1964), and measuring complexity changes across these periods may be revealing. Other scripts could be added to the current dataset, including scripts written on stone, bamboo, silk, and wood during the Zhou dynasty (1046–256 bce), clerical script (隸書), and a variety of cursive and semi-cursive scripts known from the Zhou dynasty on. Individuating and enumerating historical scripts is unavoidably subjective, but an upper bound on the number that might be considered is given by Yu Yuanwei (6th century ce), who listed around 100 script styles, many of which were ornamental and never in everyday use (Tseng, 1993).

Wherever possible, each form in an extended database should be annotated with the estimated date of production, means of production (e.g. carved in stone) and the genre of the text from which the form was collected. Compiling such a database would require a major effort from a large team of researchers, but would allow analyses of historical change that attempt to control for genre and means of production. For example, the “Chinese Calligraphy and Inscription Collection” (United Digital Publications, 2005) offers an opportunity to study changes in calligraphic styles used by poets between ca. 2205 bce and 1636 ce, while controlling for genre and medium. Regardless of how carefully an extended database is compiled, large gaps are inevitable. For example, oracle bone texts belong to a relatively narrow genre, and there is little evidence about how characters were written at the time outside the context of divination. Despite these gaps in the historical record, the available data seem sufficient to allow robust tests of Qiu’s claim that instances of complexification “pale in significance when compared with the importance of simplification” (Qiu, 2000, p. 48).

As suggested earlier, accounts of the evolution of Chinese often include several distinct changes under the broad heading of simplification (Qiu, 2000). Our work suggests an alternative approach that attempts to isolate different factors (e.g. visual simplicity, distinctiveness and compositionality) that influence the ease of reading, writing, and learning characters, and to explore ways in which these factors either support or trade off against each other. We made a start in this direction by working with formal measures of simplicity and distinctiveness, but future work can aim to extend and improve these measures, and to measure and evaluate the role of additional factors. Characterizing the factors in question is somewhat challenging, but exploring trade-offs between these factors may turn out to be even more challenging. For example, future work should aim to test the idea that attested scripts achieve near-optimal tradeoffs between simplicity and distinctiveness. Addressing this question will probably require comparing attested tradeoffs with the tradeoffs achieved by a large space of hypothetical scripts, and characterizing these hypothetical scripts is likely to require a sophisticated computational approach.

### Conclusion

Historical changes in written Chinese have undoubtedly been shaped by multiple factors, but our findings nevertheless suggest that modern characters are more complex than their oracle bone equivalents. This result can be explained in part by a trade-off between simplicity and distinctiveness, and written Chinese therefore provides yet another example of how linguistic systems are shaped by competing functional constraints. Although our work challenges the specific claim that writing systems naturally become simpler over time, it is entirely compatible with the broader view that writing systems are fundamentally shaped by the need for efficient communication.

## ACKNOWLEDGMENTS

We thank Sven Osterkamp and Wolfgang Behr for drawing our attention to important commentaries on Chinese writing, and Anthony Garnaut, Terry Regier and Yang Xu for comments on the manuscript. This work was supported in part by ARC FT190100200.

## AUTHOR CONTRIBUTIONS

SJH compiled the data, and SJH and CK wrote code for the project. SJH, PK and CK wrote the paper. All authors discussed the models and analyses and commented on the manuscript.

## Notes

^1^ Code and data are available at https://github.com/cskemp/chinesecharacters.^2^ The preregistration is available at https://aspredicted.org/x76et.pdf.
