# Supplementary material for: Simplification Is Not Dominant in the Evolution of Chinese Characters
Source: Open Mind (Camb). 2022 Dec 2;6:264–79. doi: 10.1162/opmi_a_00064 (PMC9987343; doi:10.1162/opmi_a_00064)
Supplement: Supplementary file 1 [file opmi-06-264-s001.pdf]

Supplementary Material

Simon J. Han, Piers Kelly, James Winters, and Charles Kemp

1 Character data **2**
1.1 Image preprocessing 3

2 Analysis of complexity through time **4**

3 Behavioral experiment **5**
3.1 Participants 5
3.2 Materials 6
3.3 Procedure 6
3.4 Results 7

4 Pictographic and single-component characters **11**

5 Frequency analysis **12**
5.1 Analysis of adjusted frequencies 12
5.2 Analysis of raw frequencies 14

6 Distinctiveness **16**
6.1 Changes in distinctiveness and complexity 17
6.2 Statistical analysis of distinctiveness 18
6.3 Frequency and distinctiveness 19

7 Alternative complexity measures **21**

1 Character data

Oracle, bronze and seal script forms were scraped from `hanziyuan.net`. According to Richard Sears (Sears, Richard, 2011), the main developer of `hanziyuan`, the original sources for these three collections of forms are Xu jiaguwen bian (甲骨文), Jinwen bian (金文编), and the earliest complete copy of the Shuowen jiezi (文解字). Other online databases of historical characters are available, including the Multi-function Chinese Character database (`humanum.arts.cuhk.edu.hk/Lexis/lexi-mf/`) and the Xiaoxuetang Wenzixue database (`xiaoxue.iis.sinica.edu.tw/`). Both databases draw on a broader range of sources than `hanziyuan` and include more extensive documentation, and are therefore preferable for some purposes. We chose to work with `hanziyuan` because this site is organized in a way that made it relatively straightforward to compile a large dataset for computational analysis, and because the character images on this site have clean backgrounds that are relatively uniform in quality.

To scrape all forms from `hanziyuan` we sent a HTTP request form for each character in the CLD to an endpoint provided by the website. The response packet contained a list of svg images and accompanying script labels encoded in base64. These images were decoded and then saved in .svg format.

Handwritten forms were sourced from the Traditional Chinese Handwriting Dataset (Chen, 2020) and the CASIA Offline Chinese handwriting database (Liu et al., 2011).[1] The CASIA database is divided into Training and Test sets, and we used only images from the Test set. These datasets provided us with approximately 50 traditional forms and 130 simplified forms for each character. There are some stylistic differences between the data sets – in particular, the traditional forms tend to have thinner strokes and are more subject to aliasing, and the simplified forms tend to be more cursive than the traditional forms. Figure S12 gives some sense of the stylistic variation between the two data sets.

To link traditional characters with images from other scripts, we needed to establish a mapping between simplified and traditional characters. In some cases multiple traditional characters correspond to the same simplified character, and we aimed to work with the most typical traditional version of each simplified character. To achieve this goal, we linked each simplified character with the traditional character specified by the simplified → traditional mapping included in the `opencc` package, and dropped all traditional characters that were not linked in this way.

Table S1 shows the number of characters in our data set for each script and for each stream. The streams used to analyze changes in complexity (Figure 2) and distinctiveness (Figure 2) are different because for distinctiveness it is

[1] We used the version of the CASIA database available at `https://www.kaggle.com/pascalbliem/handwritten-chinese-character-hanzi-datasets`.

Script	Total	**Complexity streams** (Figure 2)				**Distinctiveness streams** (Figure 6)			
		Orcl	Brz	Seal	Trad	Orcl	Brz	Seal	Trad
Oracle	651	651				491			
Bronze	1128	517	611			491	578		
Seal	2905	618	580	1707		491	578	1692	
Traditional	3835	647	609	1694	885	491	578	1692	857
Simplified	3852	648	610	1702	857	491	578	1692	857

Table S1: Numbers of characters in each script and stream. The Traditional and Simplified scripts both come in three versions (handwritten, Hiragino Sans GB and SimSun), and the same characters are included in all three versions.

important to ensure that each stream has the same number of characters for each script. The Oracle distinctiveness stream was therefore created by starting with the Oracle complexity stream and dropping all characters that were missing from at least one script, leaving 491 characters that appear in all 5 scripts. The remaining distinctiveness streams were created analogously.

1.1 Image preprocessing

To prepare our dataset for analysis, each image was first converted into .png format and then binarised, cropped, skeletonised, and scaled to a standard size. Each image was put through an identical preprocessing pipeline in order to account for differences in scaling and brush thickness that may have otherwise biased our complexity measures.

To binarise images, we converted any pixel that was not white into a black pixel. We then cropped each image such that the leftmost, rightmost, topmost and bottommost black pixels touched the edge of the image. Images were then padded with white pixels to be square (either top and bottom padding or right and left padding, but not both) and rescaled to be 290 by 290 pixels in size. We then added a white boundary around the entire image, producing an image of 300 by 300 pixels. To control for stroke thickness, we next skeletonized the image so that each stroke was exactly 1 pixel wide. Characters which originally had wide strokes end up relatively small after skeletonization, so we repeated the cropping, padding, rescaling and skeletonization sequence one more time to end up with the final image. Each processing step was carried out using the `scikit-image` package in Python.

Figure S1 shows all images from Figure 1 after they have passed through the image preprocessing pipeline. The skeletonization step introduces image artifacts in some cases: for example, the processed bronze form for 山 has small spines on the left and the right. These spines, however, are difficult to automatically distinguish from small strokes that are genuinely part of the character — e.g. the small strokes visible in the bronze form for 車. We therefore did not include any processing step that aimed to remove these spines.

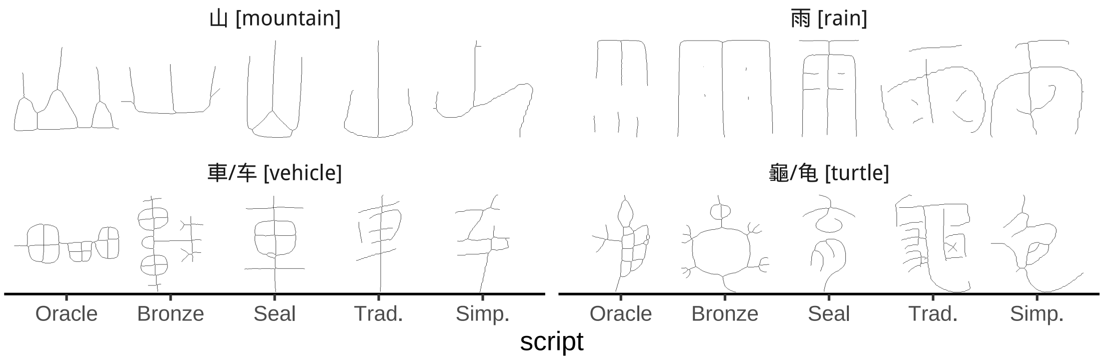

Figure S1: Forms from Figure 1 after passing through the image preprocessing pipeline.

2 Analysis of complexity through time

For each image we computed perimetric complexity using Justin Sulik's implementation[2] of an algorithm described by Pelli and collaborators (Pelli et al., 2006).

To analyze changes in complexity through time, we used the `brms` package with default priors[3] to run a Bayesian mixed effects regression with script as a predictor of complexity, and included character as a random intercept and a random slope for script. We treated script as an ordered factor, and because we are interested in differences between successive scripts, we used successive difference coding for this factor. Posterior distributions on the coefficients that capture differences between successive scripts are shown in Figure S2. All of these distributions are far from zero, suggesting that there are are robust increases in complexity from each of the first two scripts to the next, and robust decreases in complexity thereafter.

[2]Available from `github.com/justinsulik/pythonScripts/`

[3]By default brms uses improper flat priors on the coefficients of fixed effects, and a half student-t prior with 3 degrees of freedom, a location of 0 and a scale of 2.5 on the intercept and on the standard deviations of the random effects.

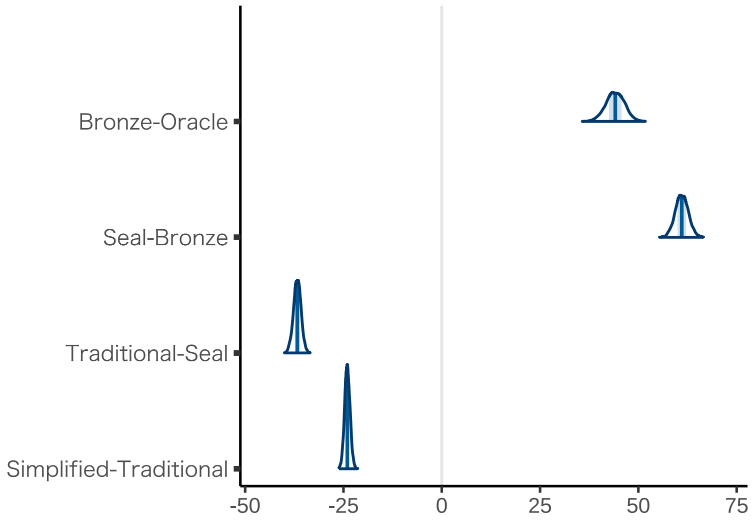

Figure S2: Posterior distributions for regression coefficients that capture differences in complexity between successive scripts. The model formula was complexity $\sim$ script + (1+script|character) .

3 Behavioral experiment

After carrying out the computational analysis summarized by Figure 2, we preregistered an experiment designed to test the hypothesis that people would rate traditional pictographic characters as more complex than their oracle equivalents.[4] The experiment included two conditions that differed according to whether the traditional characters shown were handwritten or printed, and was carried out with approval from Human Ethics Committee LNR 3D at the University of Melbourne.

3.1 Participants

For each condition, 200 participants were recruited from Prolific and paid £1.20 for a 10 minute task. Because we aimed to elicit judgments of visual complexity that were relatively unaffected by prior linguistic knowledge, the study was restricted to participants who were not native speakers of Mandarin, Cantonese or Japanese or fluent in at least one of these languages. In each condition, the response of one participant was not recorded because of server error, leaving 199 responses per condition.

[4]The preregistration is available at `https://aspredicted.org/x76et.pdf`

3.2 Materials

In addition to the 155 pairs of pictographic characters, we included 5 check pairs: (七,乇), (也,匜), (上,卡), (元,刓) and (只,咫). In each case the first member of the pair corresponds to a subset of the second member, and is therefore unambiguously simpler. For the handwritten condition, all check characters were drawn from the Traditional Chinese Handwriting dataset. For the printed condition, one check character in each pair was drawn from this dataset and the other was shown in Hiragino Sans GB.

As for our computational analyses, all images were scaled to a standard size of 300 by 300 pixels. Unlike the computational analyses, no skeletonization was applied because we wanted the characters to look as natural as possible.

3.3 Procedure

The task introduction stated that "Chinese characters have changed over time," and that "some characters have become more complex over time" and others have "become simpler over time." Participants then learned that they would see pairs of characters, and would have to "decide which character in each pair is more complex." Responses were provided on a 6 point scale where L3, L2 and L1 indicated that the left character was "much more complex", "moderately more complex" or "slightly more complex" than the right character, and R1, R2, and R3 were defined analogously.

After learning about the response scale, participants were shown four versions of the same character, two printed in different fonts, and two handwritten. Participants were told that the versions have "different styles and stroke thicknesses, but the underlying character is the same in all cases", and that all four versions were therefore equal in complexity. When comparing characters, participants were instructed to "ignore superficial differences in style and stroke thickness" and to "focus instead on the underlying shapes of the characters." Participants were also told that some of the characters had been scanned from printed sources, and that the digitization process had introduced some imperfections which should be ignored.

After the introduction, participants were given a multiple choice test with three questions designed to ensure that they had understood the task and the response scale. Participants who answered any of the questions incorrectly were sent back to the beginning of the experiment, and continued looping through the instructions until they could answer all three questions correctly.

Each participant then provided ratings for a random set of 50 pictographic characters and for all 5 check

pairs. Presentation order was randomized within participants. The left-right arrangement of each pair was also randomized within participants, so that oracle forms sometimes appeared on the left and sometimes appeared on the right.

After finishing the task, participants were asked to rate how much they knew about Chinese characters on a 4 point scale ("A lot", "some", "a little", "virtually nothing"). These ratings were not analyzed and will not be subsequently discussed.

3.4 Results

The preregistered inclusion criterion specified that participants would be included in the analysis only if they responded correctly to all 5 check pairs. In the two conditions, 170 out of 199 (handwritten) and 132 out of 199 participants (printed) met this criterion. The difference in inclusion rates across conditions probably reflects the fact that each check pair in the printed condition included one printed form. In the printed condition only, participants with a strong tendency to treat printed forms as either more or less complex than handwritten forms would have been likely to respond incorrectly to at least one check pair.

For our analyses, we mapped the 6 point response scale to a scale from -2.5 to 2.5. Zero on this scale indicates that oracle and traditional forms of a character are equally complex. Mean responses for each character are plotted in Figure 3b, and distributions of responses for each character are shown in Figures S3 and S4. Character means for the two conditions were highly correlated (r = 0.91). Figure S5 shows that ratings in both conditions were also correlated with changes in complexity as measured using perimetric complexity. For the printed condition, the y-intercept of the regression line in Figure S5 is negative, suggesting that perimetric complexity tends to regard printed characters as more complex than people do. There is no similar discrepancy for the handwritten condition.

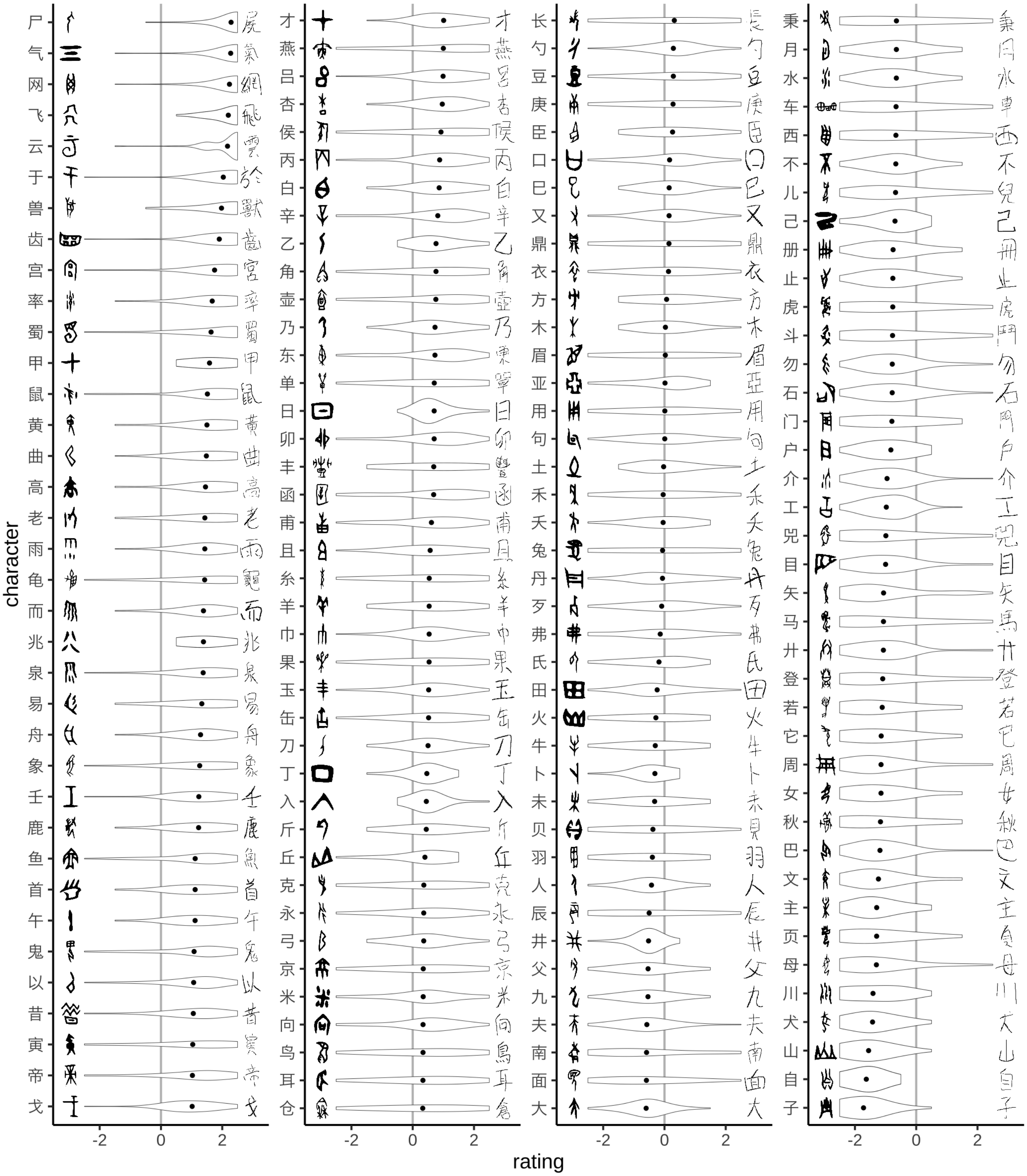

Figure S3: Ratings distributions for individual characters in the handwritten condition.

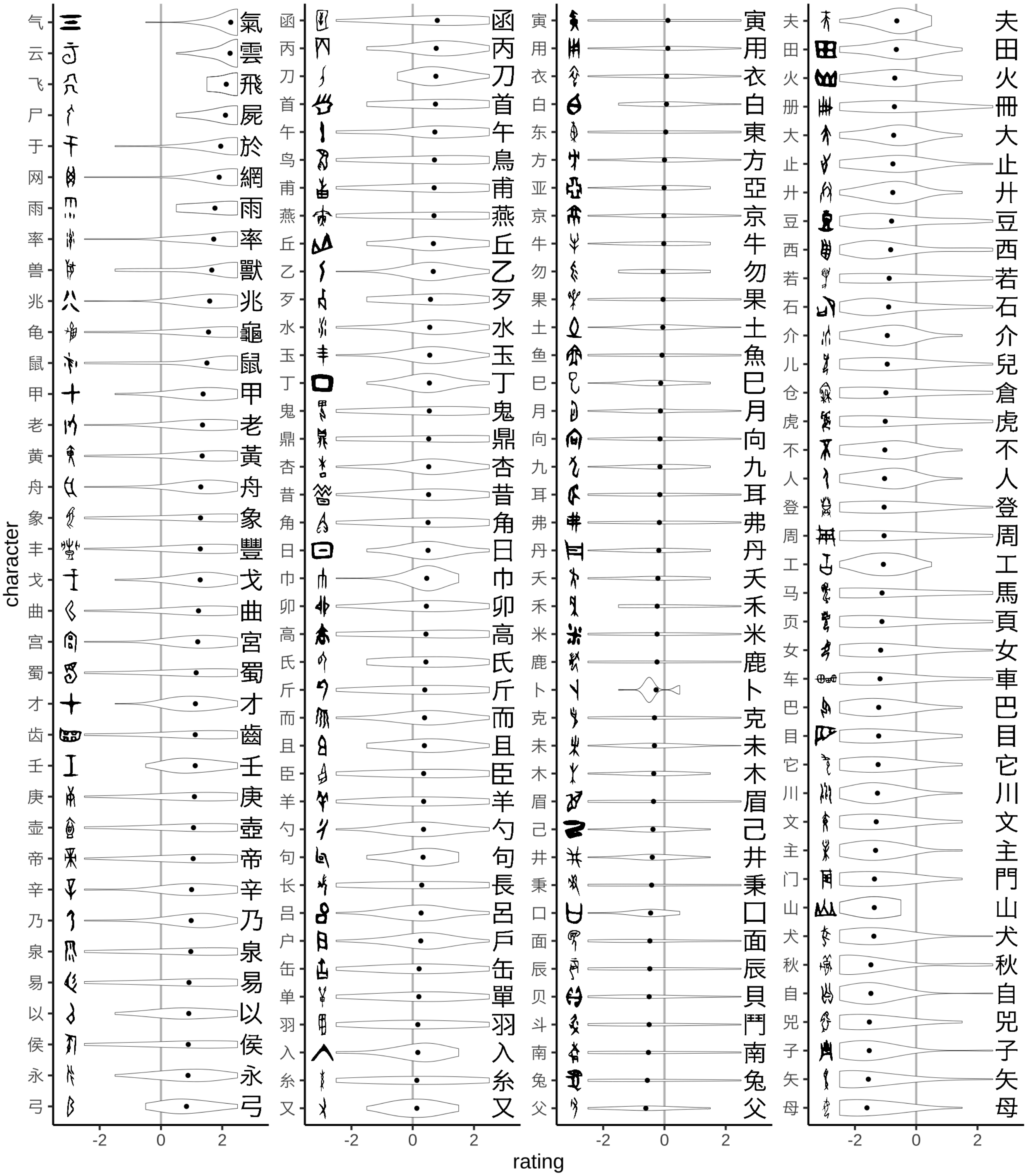

Figure S4: Ratings distributions for individual characters in the printed condition.

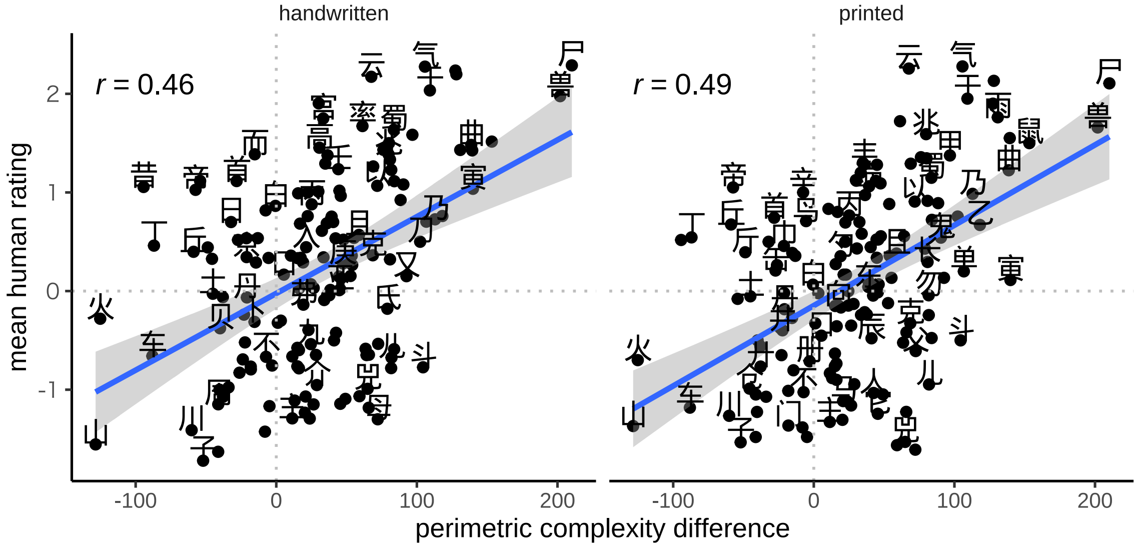

Figure S5: Human ratings of relative complexity against changes in perimetric complexity.

For the preregistered statistical analyses, we converted each response to a binary complexification variable that indicates whether the traditional form of a given character was rated as more or less complex than the oracle form. We ran a mixed effects logistic regression model with complexification as the dependent variable and character and participant id as random effects. Table S2 shows results for the handwritten condition when the full model is compared to a null model with zero intercept. The intercept of the full model was significantly different from 0 (β_0 = 0.51, 95% CI = [0.11, 0.90], $\chi^2(1)$ = 6.3, $p < 0.05$), and this intercept indicates that the overall probability that a traditional character is rated more complex than its oracle equivalent is 0.62. The p value reported is based on a likelihood ratio test comparing the full and null models.

For the printed condition, the same analysis yielded an intercept that was not significantly different from 0 (β_0 = 0.19, 95% CI = [-0.28, 0.65], $\chi^2(1)$ = 0.62, $p \approx 0.43$), and this intercept corresponds to an overall probability of 0.55 that a traditional character is rated more complex than its oracle equivalent. The comparison between full and null models for the printed condition is shown in Table S3.

model	AIC	BIC	Chisq	Df	Pr(>Chisq)
$0 + (1 \vert \text{character}) + (1 \vert \text{participantID})$	7578.45	7592.54			
$1 + (1 \vert \text{character}) + (1 \vert \text{participantID})$	7574.14	7595.28	6.31	1	0.012

Table S2: Comparison between models with and without an intercept. The dependent variable is human ratings of the complexity of handwritten traditional characters relative to Oracle forms.

We also fit the same logistic regression model using the `brms` package with default priors. The 95% credibility

model	AIC	BIC	Chisq	Df	Pr(>Chisq)
0 + (1|character) + (1|participantID)	5750.77	5764.36			
1 + (1|character) + (1|participantID)	5752.16	5772.54	0.62	1	0.432

Table S3: Comparison between models with and without an intercept. The dependent variable is human ratings of the complexity of printed traditional characters relative to Oracle forms.

intervals on the intercept were [0.11, 0.89] (handwritten) and [-0.24, 0.66], suggesting support for our hypothesis only in the handwritten condition.

One reason why the handwritten condition produced stronger results than the printed condition may be that some participants used symmetry and regularity as cues to simplicity, which favor printed forms relative to handwritten forms. Although the results in the printed condition were not statistically significant, both conditions produced an effect in the same direction, suggesting overall support for the hypothesis that traditional forms are more complex than oracle forms.

4 Pictographic and single-component characters

We used two Bayesian regression models to explore the increases in complexity shown in Figures 3a and 3b. The dependent variable in both cases was complexification, where complexification is defined as the difference in complexity between the traditional and the oracle bone scripts. The first regression model included character type as a categorical predictor and a zero intercept. Posterior distributions on the regression coefficients are shown in Figure S6a. The distributions for pictologic and other characters barely overlap with zero, and the distributions for the three other classes are far from zero, suggesting that there is a reliable increase in complexity for all classes.

The second regression model was similar but included number of components as a categorical predictor. We treated number of components as categorical because we are interested in whether there is a reliable increase in complexity for each level of this variable, and not especially interested in whether complexification increases as the number of components increases. The posterior distributions on the regression coefficients in Figure S6b suggest that there is a reliable increase in complexity for all numbers of components, including for characters with a single component.

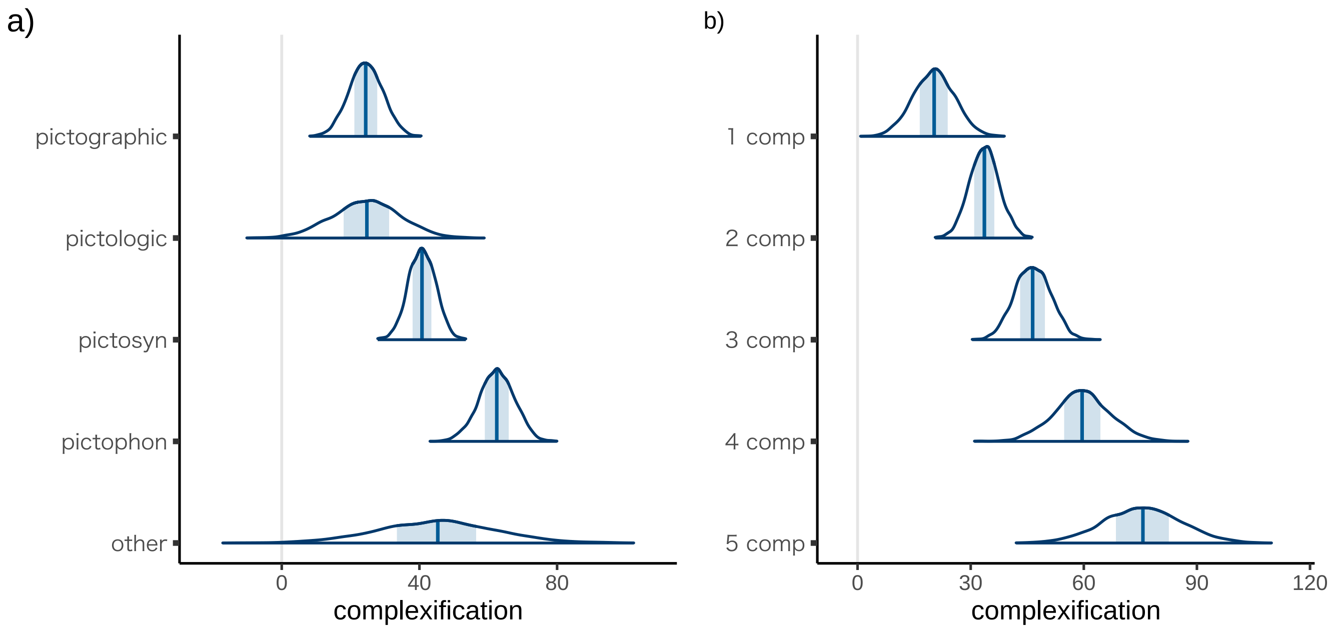

Figure S6: Posterior distributions for regression coefficients that capture the extent to which different classes of characters complexify from the oracle bone script to the traditional script. The model formulae were (a) complexification $\sim$ 0 + character_type; (b) complexification $\sim$ 0 + number_of_components.

5 Frequency analysis

5.1 Analysis of adjusted frequencies

Figure 4 shows that perimetric complexity tends to increase as the frequency of characters decreases, and also suggests an interaction between frequency and script. Here we present a statistical analysis that supports these conclusions. We work here with log frequencies (base 10), and for ease of interpretation we invert log frequency to create a variable called `rarity` (as rarity increases, log frequency decreases). For ease of visualization, Figure 4 makes use of frequency bins, but no binning is used for the analyses that follow.

We used the `brms` package to run a Bayesian regression that included both script, rarity and an interaction between script and rarity as predictors of complexity. Posterior distributions on the regression coefficients are shown in Figure S7. Most distributions are far from zero, confirming that complexity changes reliably through time, and that rarer characters tend to be more complex and to change in complexity faster than more common characters. One distribution, however, is centered on zero, suggesting that the transition between traditional and simplified scripts provides no evidence for an interaction between rarity and script. This result may reflect the fact

that the transition between traditional and simplified scripts resulted from a government decree instead of emerging from a more organic process of change.

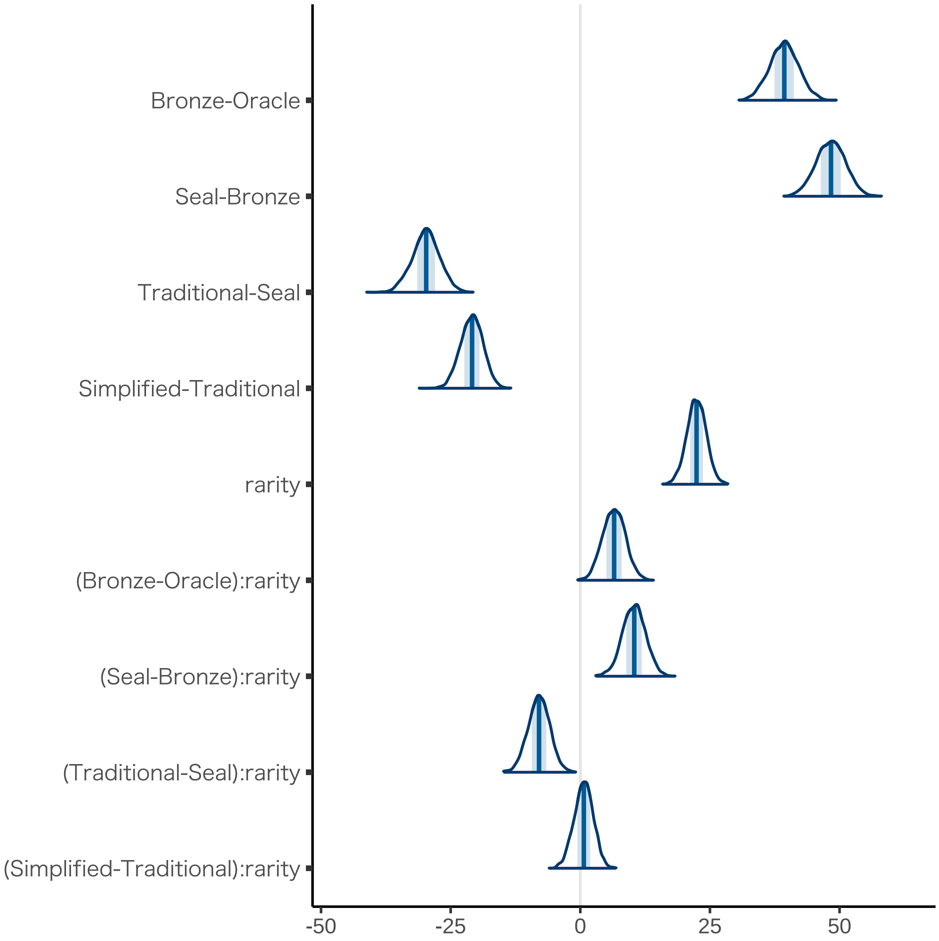

Figure S7: Posterior distributions on regression coefficients for model that includes script and rarity as predictors along with an interaction between script and rarity. The model formula was complexity $\sim$ script + rarity + script:rarity + (1+script|character) .

Because the results summarized by Figure S7 do not enable a direct comparison between oracle and traditional scripts, we repeated the analysis including data from only these scripts. The posterior distributions in Figure S8 support the conclusion that complexity showed a net increase between the oracle and traditional scripts, and that rare characters are both more complex and showed a greater increase in complexity.

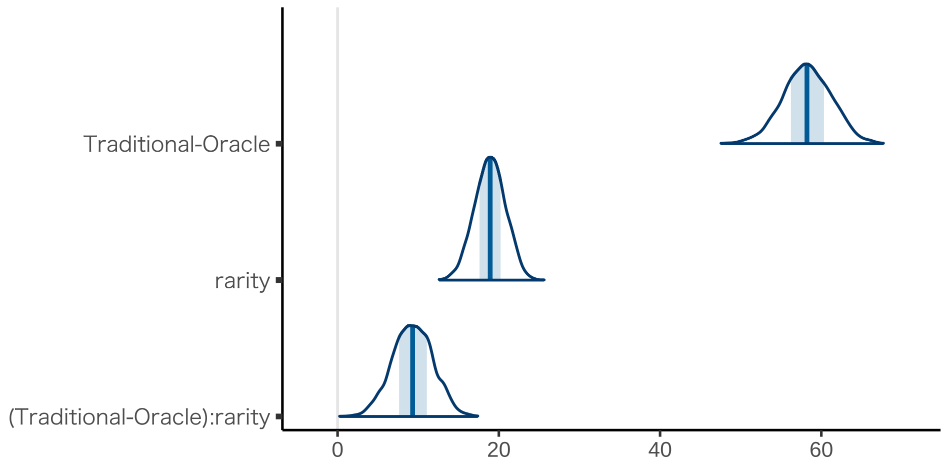

Figure S8: Posterior distributions on regression coefficients for analysis similar to Figure S7 that includes oracle and traditional scripts only.

5.2 Analysis of raw frequencies

Some characters are components of other characters, and the frequencies used so far were adjusted so that a character's frequency is based in part on the number of times it is written as part of another character. This section repeats our analyses of frequency using raw rather than adjusted frequencies. Figure S9 shows the relationship between script, raw frequency, and complexity.

The Bayesian analysis in Figure S10 shows that the distributions for all four interaction coefficients now overlap with zero. This result therefore suggests that working with raw frequencies yields relatively weak evidence of an interaction between rarity and script. A similar conclusion emerges from the analysis that considers the oracle and traditional scripts only. Figure S11 supports the conclusion that complexity shows a net increase between these two scripts and that rarer characters tend to be more complex, but does not suggest that rarer characters show greater increases in complexity.

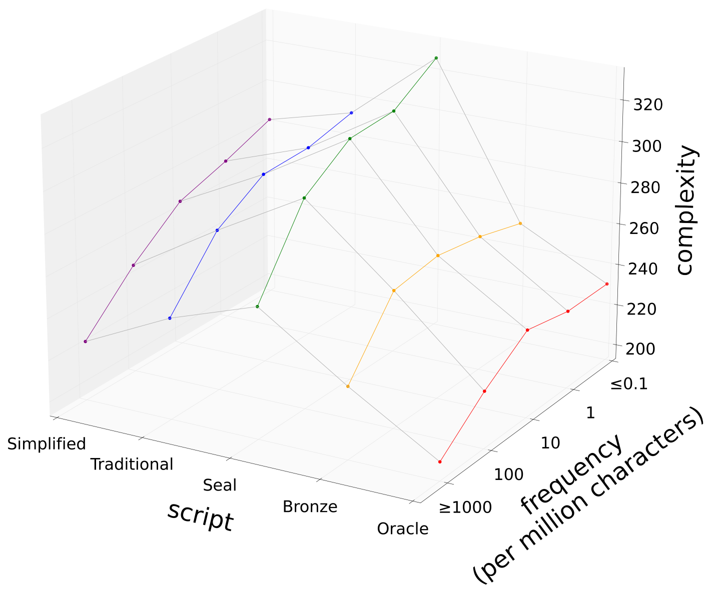

Figure S9: A version of Fig 4 based on raw frequency rather than adjusted frequency.

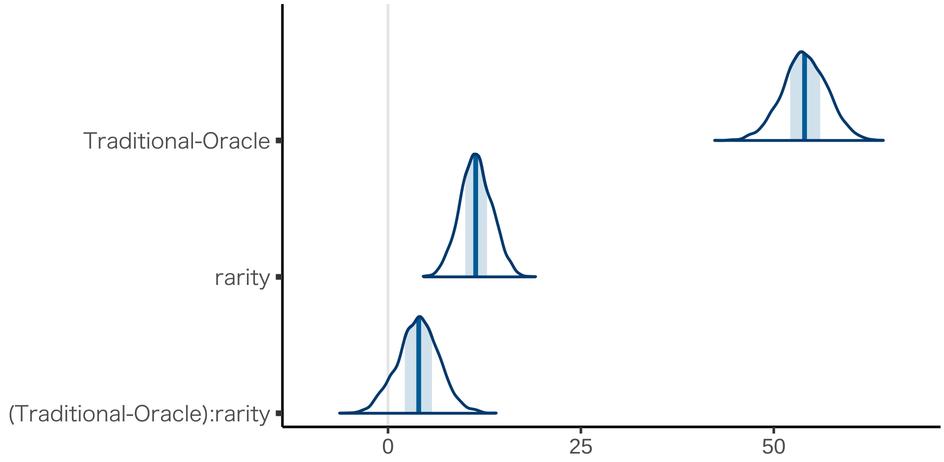

Figure S11: Posterior distributions corresponding to Figure S8 when rarity is based on raw rather than adjusted frequency.

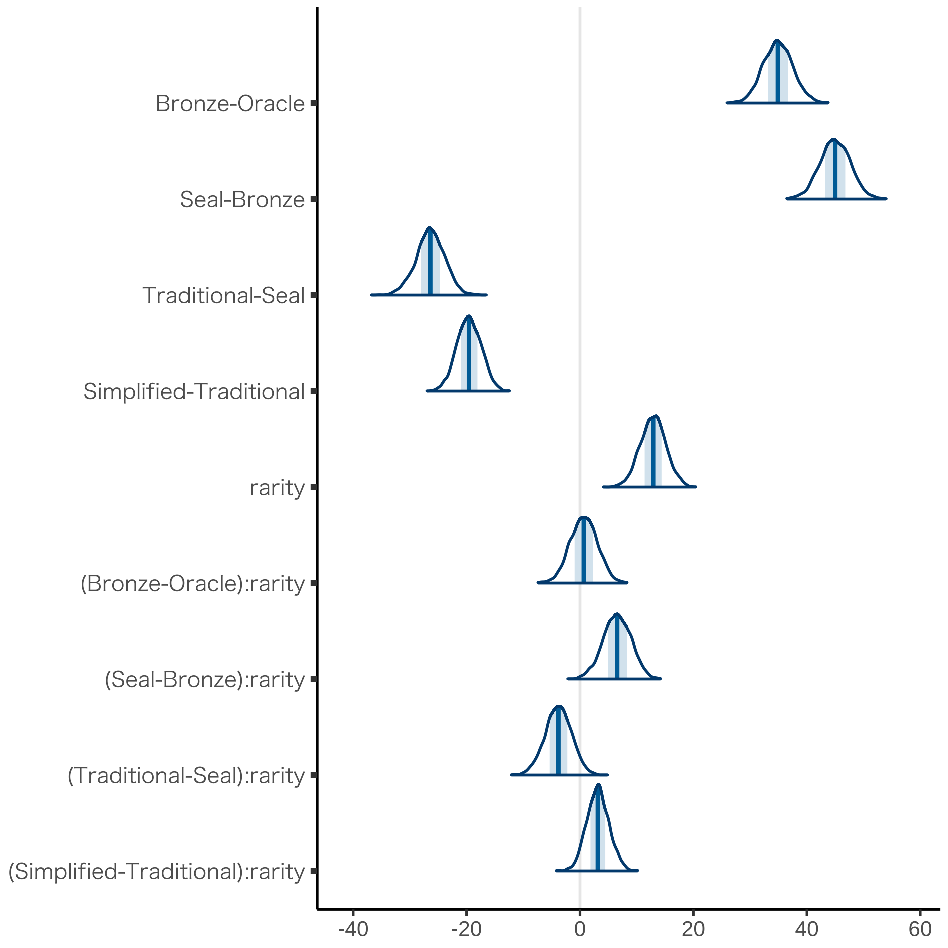

Figure S10: Posterior distributions corresponding to Figure S7 when rarity is based on raw rather than adjusted frequency.

6 Distinctiveness

Our distinctiveness analyses used a pretrained GoogLeNet network available from `https://github.com/chongyangtao/DeepHCCR`. The network was trained on images from the CASIA database, which is also the source of the simplified characters in our dataset. For the distinctiveness analyses only, we added an additional step to the image processing pipeline that dilated each character skeleton by a disk of radius 5 pixels. Dilating the skeletons in this way increases the width of the strokes, and leaves the processed images more comparable to the images used to train the network.

The representations used in our analyses were drawn from layer fc1 of the network, which holds an activation vector of length 1024 that is ultimately passed through a softmax function to generate the network output. Because

the output layer classifies each image as one of 7354 simplified characters, the penultimate layer is a natural choice for an analysis that includes images from different scripts.

All distinctiveness analyses used a single representative of each character in each script, and we chose the form with median perimetric complexity as this representative. Distinctiveness is defined as the average Euclidean distance between a character and its 20 nearest contemporary neighbours. Figure S12 shows the form for 天 [sky] in each script, along with its 10 nearest neighbors. Although the network is trained on simplified characters only, it performs reasonably well at identifying characters with similar shapes in all scripts.

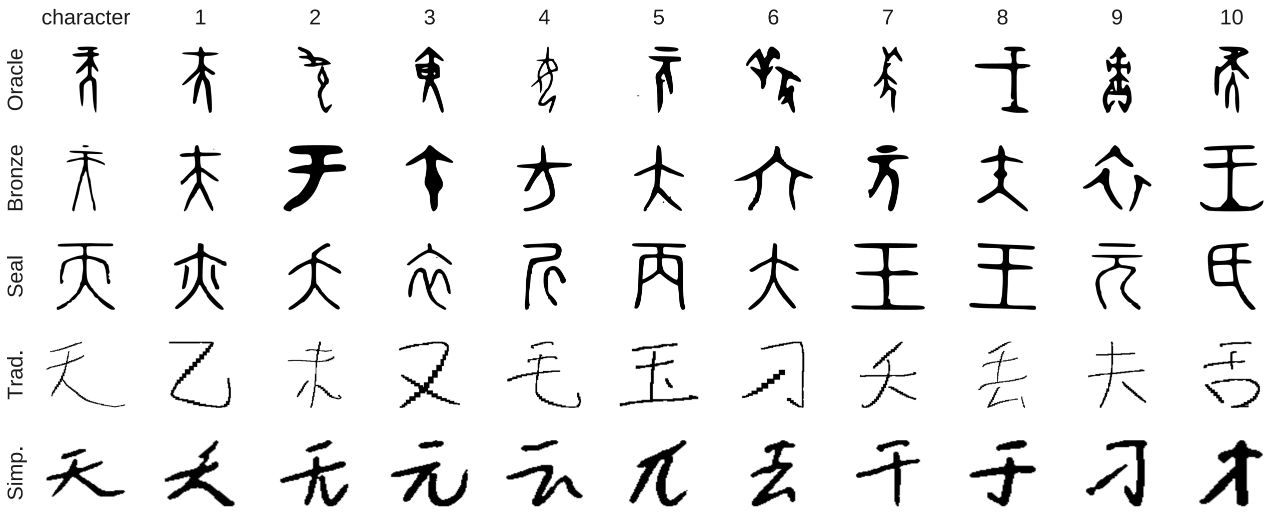

Figure S12: Nearest neighbors of 天 [sky] according to our CNN-based distance metric. The leftmost column shows the form for 天 within each script, and the remaining columns show the ten most similar contemporary forms.

6.1 Changes in distinctiveness and complexity

The results in the main text strongly suggest that changes in distinctiveness are correlated with changes in complexity. To confirm this conclusion we compared changes in distinctiveness and complexity between the oracle and traditional scripts for all characters belonging to the oracle stream in Figure 6b. As expected, Figure S13 shows that the characters that increase most in complexity also tend to be the characters that increase most in distinctiveness.

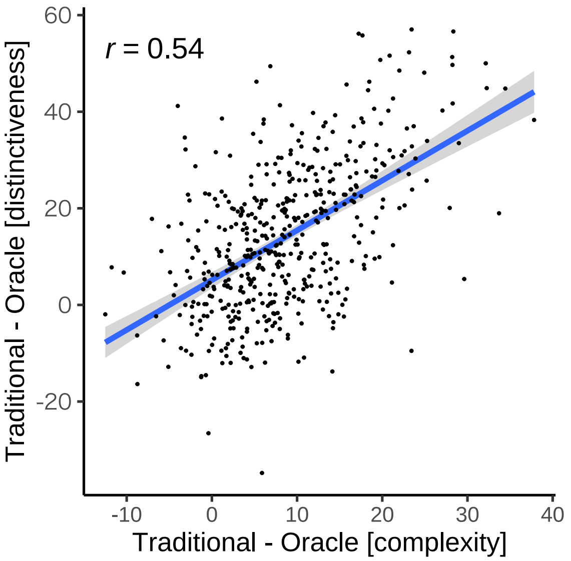

Figure S13: Changes in distinctiveness are correlated with changes in complexity. Each point represents a character from the oracle stream, and the axes show changes in distinctiveness and perimetric complexity between the oracle and traditional scripts (positive values indicate increases in distinctiveness and complexity).

6.2 Statistical analysis of distinctiveness

To analyze the changes in distinctiveness summarized by Figure 6b, we used the same Bayesian approach described previously but specified distinctiveness rather than complexity as the dependent variable. Posterior distributions on the coefficients that capture differences between successive scripts are shown in Figure S14. 95% credibility intervals associated with each distribution exclude zero, suggesting that there are are robust increases in distinctiveness up to the traditional script and a decrease in distinctiveness between the traditional and simplified scripts.

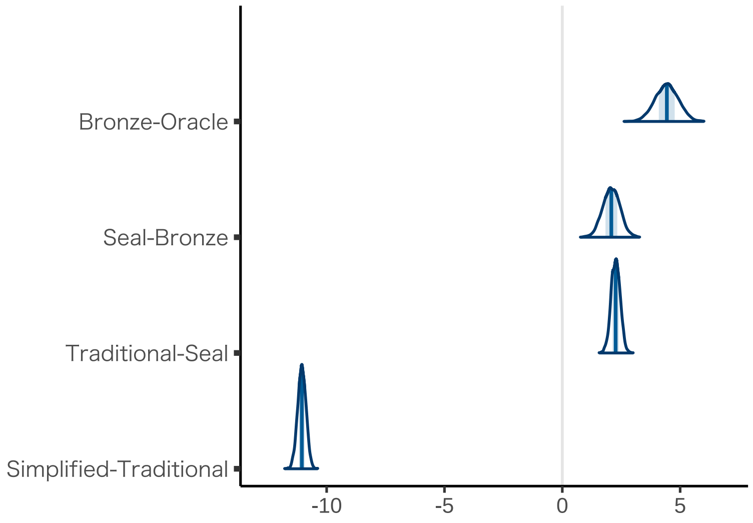

Figure S14: Posterior distributions for regression coefficients that capture differences in distinctiveness between successive scripts. The model formula was distinctiveness $\sim$ script + (1+script$|$character) .

6.3 Frequency and distinctiveness

Figure 4 suggests that changes in complexity over time are qualitatively similar for characters in different frequency bins, and we asked whether changes in distinctiveness would also be similar for characters of different frequencies. Figure S15 is directly analogous to Figure 4 except that it shows changes in distinctiveness rather than complexity. The figure is based on characters from the Oracle stream in Figure 6b, which means that a single set of characters is analyzed across all scripts and that the results for different scripts can be directly compared.

For characters in 5 of the 6 frequency bins, distinctiveness rises up until the traditional script and then falls, which matches the overall pattern shown in Figure 6b. For the most frequent characters, however, there is no monotonic progression over the first four scripts, and the curve seems essentially flat. We are reluctant to conclude much from this finding because the distinctiveness measure is noisy, because the bin in question includes 27 characters only, and because binning is an approach that may be useful for exploratory visualization but that discards statistical information.

To develop a statistical analysis of the relationship between frequency and distinctiveness we used the same Bayesian approach previously used to analyze frequency and complexity — and as before the analysis makes no use of frequency bins. Figure S16 shows posterior distributions on the coefficients of a regression analysis that included both script, rarity (the inverse of frequency) and an interaction between script and rarity as predictors of distinctiveness. The top four distributions in Figure S16 suggest that the changes in distinctiveness between

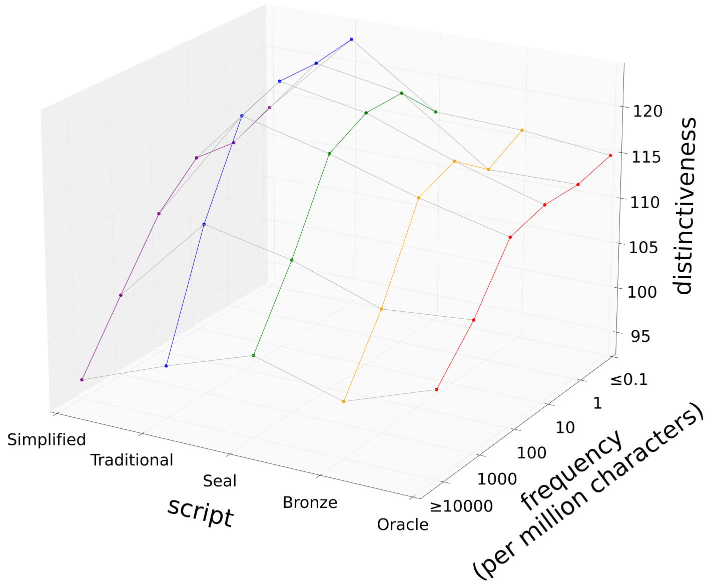

Figure S15: Distinctiveness over time for characters in six frequency bins. The labels of the frequency bins represent counts per million characters. The plot is based only on characters from the Oracle stream plotted in Figure 6b.

successive scripts are all robust, and the distribution on the rarity coefficient confirms that rarer (less frequent) characters tend to be more distinctive. The transitions from Oracle to Bronze and Bronze to Seal both provide evidence of an interaction between rarity and changes in distinctiveness, but the remaining two transitions do not.

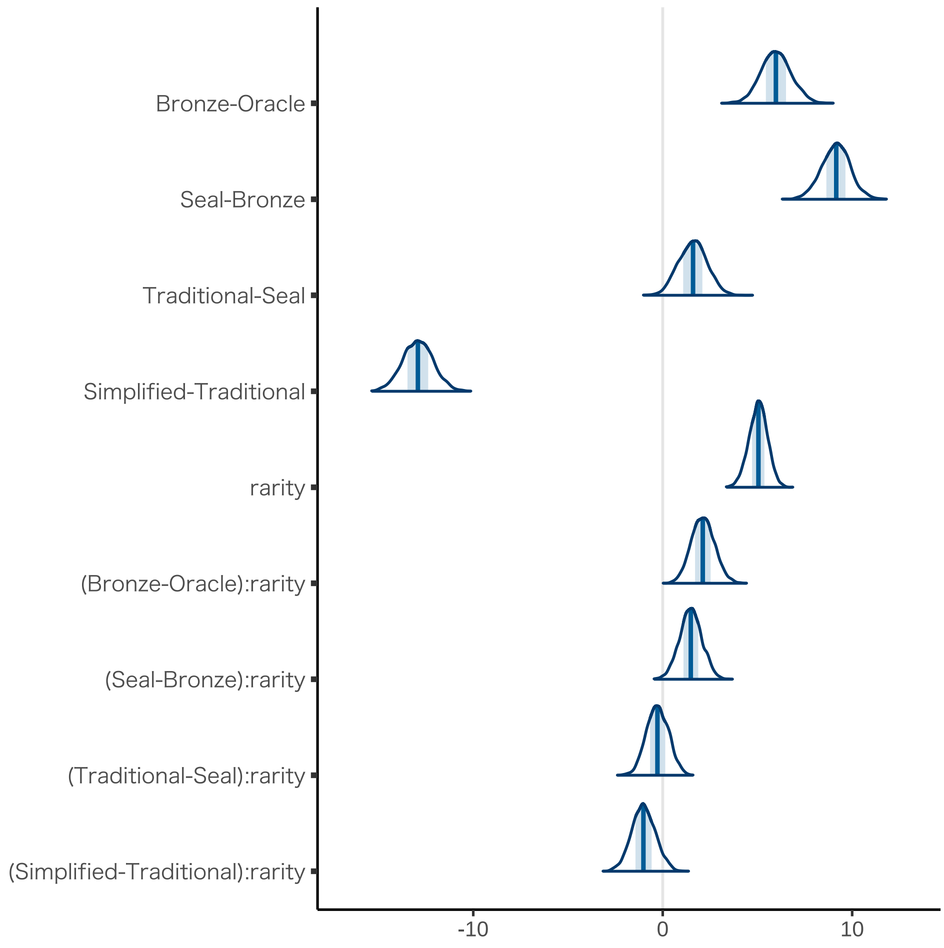

Figure S16: Posterior distributions on regression coefficients for model that includes script and rarity as predictors along with an interaction between script and rarity. The model formula was distinctiveness $\sim$ script + rarity + script:rarity + (1+script|character) .

7 Alternative complexity measures

Perimetric complexity has been widely used by previous researchers as a measure of visual complexity, and we chose it as our primary measure for compatibility with the literature. This section considers several alternative complexity measures.

After preprocessing, all of our images are black skeletons on a white background of standard size, and a simple way to measure complexity is to count the number of black pixels. Figure S17a shows that the resulting pixel-based complexity measure correlates highly with perimetric complexity.

Some work on Chinese characters uses stroke count as a measure of complexity. Figure S17b shows that

this measure is also highly correlated with perimetric complexity. The plot in Figure S17b is based on simplified characters only, because segmenting historical forms (e.g. oracle bone forms) into strokes is not straightforward.

Although we focused on visual complexity, the writing system may be shaped in part by forces that act to reduce motor complexity, or the complexity of writing characters. `zdic.net` is an online historical dictionary including animations that show how simplified characters are written. For each character, we used the number of frames in its animation as a rough measure of the time taken to write the character. Figure S17 shows that this measure of motor complexity is highly correlated with perimetric complexity.

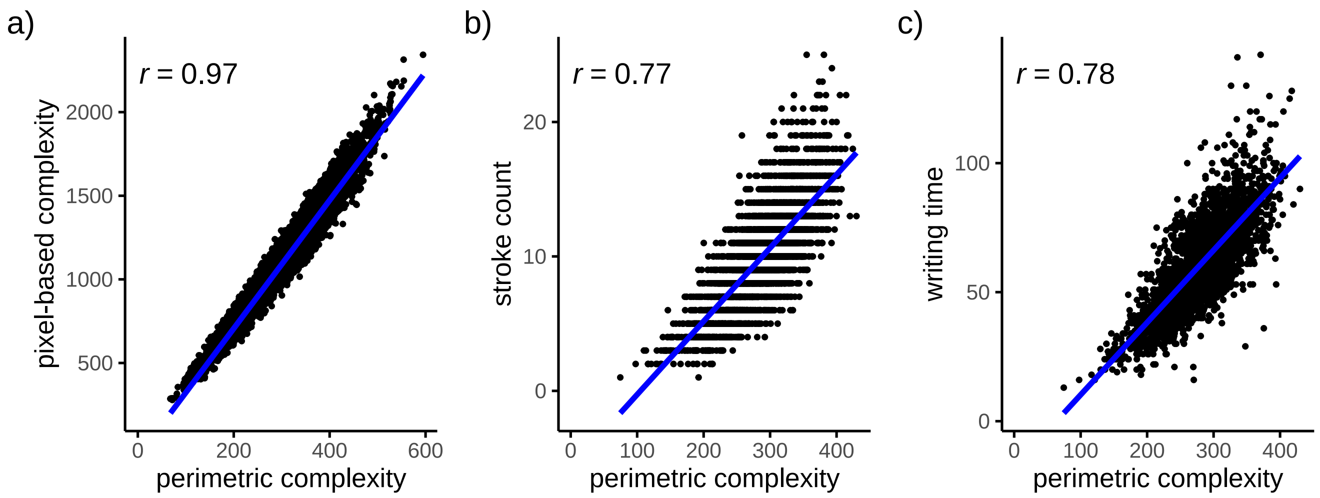

Figure S17: Alternative complexity measures plotted against perimetric complexity. Each point represents a character. The results for pixel-based complexity (a) are based on images from all scripts. The results for stroke count (b) and writing time (c) are based on simplified characters only.

Researchers from several disciplines have explored the idea that the complexity of an object corresponds to the length of its shortest description (Chater and Vitányi, 2003; Grünwald, 2007; Leeuwenberg, 1971; Simon, 1972). Inspired by this idea, previous work on the evolution of writing systems has used a measure of descriptive complexity that corresponds to the length of an image's representation in a vector graphics format (Kelly et al., 2021; Miton and Morin, 2021). We implemented the same approach by using the `potrace` tool with default options to convert each processed image into a svg (scalable vector graphics) file, and took the length of the resulting file in characters as a measure of descriptive complexity.

Within each script, Figure S18 shows that descriptive complexity and perimetric complexity are highly correlated. The regression lines, however, vary across scripts, suggesting that descriptive complexities are systematically higher for the traditional and simplified scripts than for the remaining three scripts. This discrepancy probably

results from artifacts due to digitization. Figure S1 shows that image skeletons for traditional and simplified characters tend to have strokes that are not especially smooth, and the reason is that our data for these scripts were sourced from collections of relatively low resolution images. Perimetric complexity is not especially sensitive to whether a stroke is perfectly smooth, but each small wobble or inflection increases the descriptive complexity of an image.

Although Figure S18a suggests that our measure of descriptive complexity is unreliable when comparing characters across scripts, Figure S18b shows this comparison anyway. There is a dramatic increase in complexity between the seal and traditional scripts, but for reasons just described the magnitude of this change is not plausible. Figure S18b also shows that descriptive complexity is substantially lower for font-based images than handwritten images. When compared to symbols generated by hand, fonts include straight lines and other strokes that are unnaturally smooth, which means that the descriptive complexity of fonts is extremely low.

Our descriptive complexity results for modern characters do not support strong conclusions, but it seems likely that the results for fonts and handwritten characters represent lower and upper bounds on the true quantity that we would like to estimate. In other words, if we had a dataset including high quality handwritten images of traditional and simplified characters, we expect that descriptive complexity would lie between the extremes of the range currently shown in Figure S18b.

References

Chater, N. and Vitányi, P. (2003). Simplicity: A unifying principle in cognitive science? *Trends in cognitive sciences*, 7(1):19–22.

Chen, P.-C. (2020). Traditional Chinese handwriting dataset. `https://github.com/AI-FREE-Team/Traditional-Chinese-Handwriting-Dataset`.

Grünwald, P. (2007). *The Minimum Description Length Principle*. MIT Press, Cambridge, MA.

Jin, Xiangheng (金祥恒) (1993). *Xu jiaguwen bian (*續甲骨文編 *)* . Yiwen yinshuguan.

Kelly, P., Winters, J., Miton, H., and Morin, O. (2021). The predictable evolution of letter shapes: An emergent script of West Africa recapitulates historical change in writing systems. *Current Anthropology*, pages 1–38.

Leeuwenberg, E. L. J. (1971). A perceptual coding language for visual and auditory patterns. *American Journal of Psychology*, 84(3):307–349.

Liu, C.-L., Yin, F., Wang, D.-H., and Wang, Q.-F. (2011). CASIA online and offline Chinese handwriting

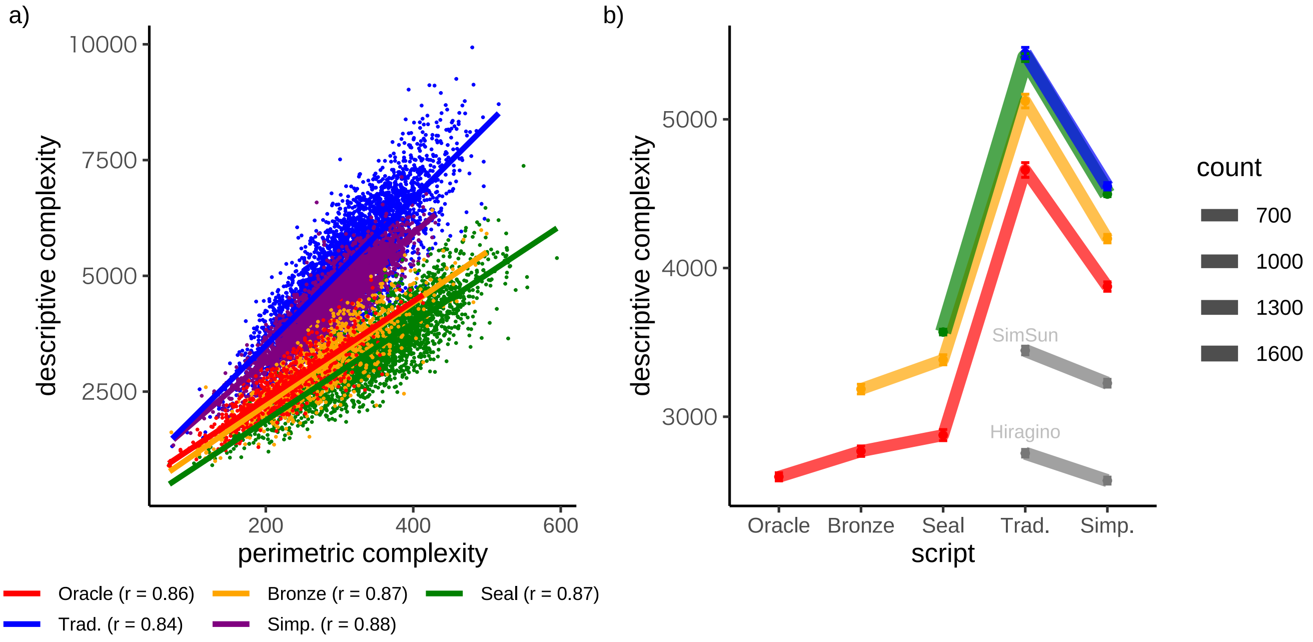

Figure S18: Analyses of descriptive complexity. (a) Descriptive complexity and perimetric complexity correlate highly within scripts, but the relationship between the two varies across scripts. (b) Changes in descriptive complexity across scripts.

databases. In *2011 International Conference on Document Analysis and Recognition*, pages 37–41. IEEE.

Miton, H. and Morin, O. (2021). Graphic complexity in writing systems. *Cognition*, 214:104771.

Pelli, D. G., Burns, C. W., Farell, B., and Moore-Page, D. C. (2006). Feature detection and letter identification. *Vision research*, 46(28):4646–4674.

Rong, Geng (容庚) (1959). *Jinwen bian (金文編)* .

Sears, Richard (2011). `https://commons.wikimedia.org/wiki/Commons:Ancient_Chinese_characters/Richard_Sears_Agreement`.

Simon, H. A. (1972). Complexity and the representation of patterned sequences of symbols. *Psychological Review*, 79:369–382.

Xu, Shen (許慎) (987). *Shuowen jiezi (説文解字)* . Copied by Xu Xuan (徐鉉).
